# Region-based and pathway-based QTL mapping using a *p*-value combination method

**DOI:** 10.1186/1753-6561-5-S9-S43

**Published:** 2011-11-29

**Authors:** Hsin-Chou Yang, Chia-Wei Chen

**Affiliations:** 1 Institute of Statistical Science, Academia Sinica, Nankang 115, Taipei, Taiwan

## Abstract

Quantitative trait locus (QTL) mapping using deep DNA sequencing data is a challenging task. In this study we performed region-based and pathway-based QTL mappings using a *p*-value combination method to analyze the simulated quantitative traits Q1 and Q4 and the exome sequencing data. The aims were to evaluate the performance of the QTL mapping approaches that were used and to suggest plausible strategies for QTL mapping of DNA sequencing data. We conducted single-locus QTL mappings using a linear regression model with adjustments for age and smoking status, and we also conducted region-based and pathway-based QTL mappings using a truncated product method for combining *p*-values from the single-locus QTL mapping. To account for the features of rare variants and common single-nucleotide polymorphisms (SNPs), we considered independently rare-variant-only, common-SNP-only, and combined analyses. An analysis of 200 simulated replications showed that the three region-based methods reasonably controlled type I error, whereas the combined analysis yielded the greatest statistical power. Rare-variant-only, common-SNP-only, and combined analyses were also applied to pathway-based QTL mappings. We found that pathway-based QTL mappings had a power of approximately 100% when the significance of the vascular endothelial growth factor pathway was evaluated, but type I errors were slightly inflated. Our approach complements single-locus QTL mapping. An integrated approach using single-locus, combined region-based, and combined pathway-based analyses should yield promising results for QTL mapping of DNA sequencing data.

## Background

Deep DNA sequencing technology provides not only a vast number of common single-nucleotide polymorphisms (SNPs) but also a vast number of rare variants for genetic and genomic research. Mapping a quantitative trait locus (QTL) using this kind of genomic data remains a challenging task. *p*-Value combination methods have been used in genetic association studies in which common SNPs were analyzed, but rare variants were excluded from those studies [1-6]. To the best of our knowledge, no previous studies have used *p*-value combination methods to analyze rare variants. When the *p*-value combination methods are applied to QTL mapping of deep DNA sequencing data, the performance of the methods and the plausible analytical strategies for dealing with common SNPs and rare variants remain unclear.

In this study we conduct region-based and pathway-based QTL mappings using a *p*-value combination method to identify genetic loci, regions, and pathways responsible for a quantitative trait using exome DNA sequencing data provided by Genetic Analysis Workshop 17 (GAW17). We propose and evaluate several analytical strategies for QTL mapping of common SNPs and rare variants in deep DNA sequencing data. In contrast to single-locus QTL mapping, region-based and pathway-based QTL mappings using a *p*-value combination method are multilocus methods that have the following merits [4-6]: (1) The biological knowledge of genes and pathways is incorporated; (2) the results are linked directly to biological function; (3) the multilocus methods may have greater statistical power; (4) the problem of multiple testing is reduced because fewer tests are conducted; and (5) the locus heterogeneity is mitigated because a group of loci are considered jointly. The aims of this study are to evaluate the type I error and statistical power associated with the proposed analytical strategies for region-based and pathway-based QTL mappings and to suggest plausible analytical strategies for QTL mapping of deep DNA sequencing data.

## Methods

### Materials

GAW17 provided a mini-exome DNA sequencing data set and 200 simulated data sets for a quantitative trait for 697 unrelated individuals. The 1000 Genomes Project donated genotype data composed of the DNA sequences of 3,205 genes, containing 24,487 autosomal common SNPs and rare variants [7]. More than 50% of the variants were rare and had a minor allele frequency (MAF) less than 0.01. Missing genotype data were imputed. Data were obtained on the age, sex, and smoking status for all subjects. Quantitative trait data were generated as normally distributed phenotypes.

While evaluating the statistical power of the QTL mapping approaches, we conducted QTL mappings using the simulated quantitative trait Q1, which was generated from a standard normal distribution with a residual heritability of 0.44. This trait was designed to be influenced by nine Q1-associated genes (*ELAVL4*, *ARNT*, *KDR*, *VEGFC*, *FLT4*, *VEGFA*, *FLT1*, *HIF1A*, and *HIF3A*), which function primarily in the vascular endothelial growth factor (VEGF) pathway. The 9 genes contained 125 SNPs, with 39 nonsynonymous common SNPs or rare variants (MAFs ranging from 0.07% to 16.5%) designed to additively influence Q1. The total effect sizes of the nonsynonymous variants within the nine genes ranged from 0.59 to 5.7.

In order to evaluate type I error associated with the QTL mapping approaches, we examined the association between the nine Q1-associated genes and the simulated quantitative trait Q4. Q4 was generated from a standard normal distribution and had a heritability of 0.70. This trait was designed to be uninfluenced by any of the genotyped exonic common SNPs and rare variants.

### Statistical methods

We analyzed 200 simulation replications. In each replication, we conducted single-locus QTL mappings using a linear regression model in which the quantitative trait (Q1 for the analysis of statistical power and Q4 for the analysis of type I error) was the dependent variable and genotype, age, and smoking status were independent variables. The analyses were performed using PLINK software [8]. The genotype was coded by using an additive effect random variable with values of 0, 1, or 2 for genotypes *AA*, *AB*, or *BB*, respectively, where *A* was the major allele. Tests for normality showed that Q1 followed a standard normal distribution, but Q4 violated a standard normal distribution in a large proportion of the simulation replications. Therefore, for each simulation replication, 1 million permutations were made and used to calculate an empirical *p*-value for a single-locus QTL mapping of Q4.

The methods that we used for our region-based and pathway-based QTL mappings were as follows. To account for the features of rare variants and common SNPs, we divided genetic markers into two categories: rare variants (MAF < 0.01) and common SNPs (MAF ≥ 0.01). The region-based and pathway-based QTL mappings combined *p*-values from single-locus association tests for rare variants and/or common SNPs in a region or pathway. Several analytical strategies were considered: (1) a rare-variant-only analysis, (2) a common-SNP-only analysis, and (3) a combined analysis that integrated two test statistics from a rare-variant-only analysis and a common-SNP-only analysis. The test statistics are provided in what follows.

We assume that there are *R* rare variants and *C* common SNPs in a region or a pathway and that *N* = *R* + *C*. We let {*p*_rare,_*_r_*, *r* = 1, …, *R*} and {*p*_common,_*_c_*, *c* = 1, …, *C*} denote the *p*-values of single-locus association tests of *R* rare variants and *C* common SNPs, respectively. In the rare-variant-only analysis, *p*-values for only rare variants were combined to calculate a truncated product *p*-value statistic (TPPS) [1-3]:(1)

where *I*[·] denotes an indicator function and constant *θ* denotes the truncation threshold of *p*-values. Similarly, in the common-SNP-only analysis, *p*-values for only common SNPs were combined to calculate a TPPS:(2)

In the combined analysis, *p*-values for all genetic markers were merged to calculate a combined TPPS:(3)

In this study, the truncation threshold of *p*-values was set at 0.05, and the empirical *p*-values of the TPPSs were obtained using a Monte Carlo procedure with 10,000 replications (refer to the “Correlated Tests” section in Zaykin et al. [1]). The problem of multiple testing was controlled by using false discovery rates (FDRs) [9], and FDR-corrected *p*-values were calculated independently for each of the three analyses. A QTL was declared if the FDR-corrected *p*-value was less than 0.05. In this study, a QTL could be a SNP mapped by a single-locus association test, a well-defined genomic region mapped by a region-based association test, or a biological pathway identified by a pathway-based association test. All the region- and pathway-based QTL mappings were conducted using software that we developed: Omnibus *p*-value Association Tests (OPATs).

In the region-based QTL mapping, the unit of a region could be a gene, an exonic region, or another predefined region. The unit used in this study was a gene region. Our pathway-based QTL mappings focused on the VEGF pathway because the nine Q1-associated genes were from this pathway. We evaluated power and type I error for single-locus, region-based, and pathway-based QTL mappings. The power of the QTL mappings was calculated as the proportion of times that an association test for trait Q1 accurately identified the true QTL of Q1 in 200 simulation replicates. The type I error for a QTL mapping was calculated as the proportion of times that a QTL mapping for trait Q4 identified the spurious QTL of Q4 in 200 simulation replicates. Note that simulation replications were removed from the power and type I error calculations if only one *p*-value remained after applying a *p*-value truncation. When we compared the power of single-locus and region-based QTL mappings, the comparisons were made within the same unit of a genomic segment; in other words, the genomic region was declared to be a QTL in a single-locus QTL mapping if any Q1-associated rare variants or common SNPs were found within that region.

## Results

**Figure 1:**
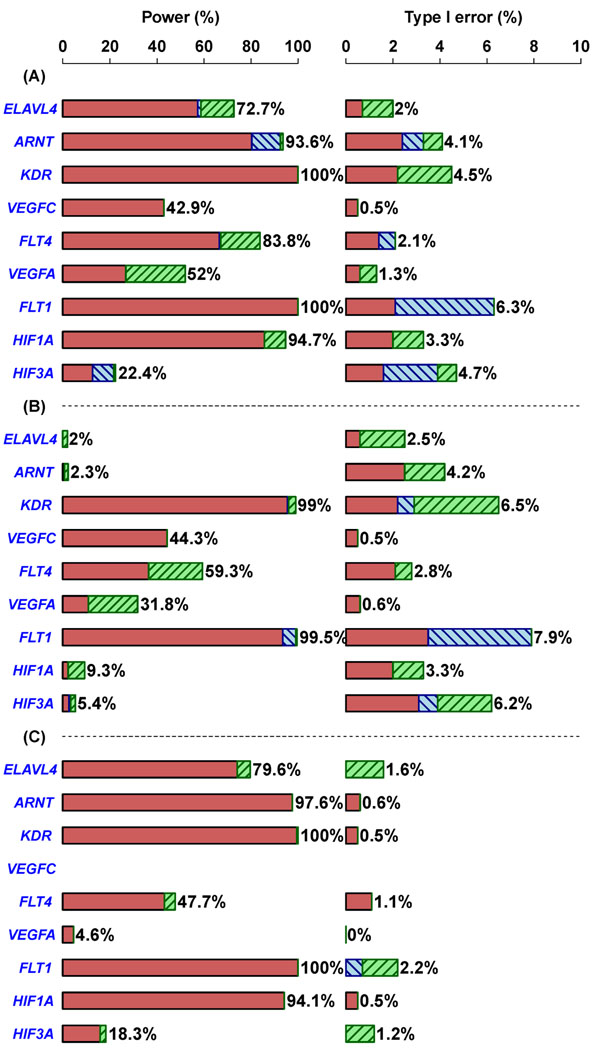
**Power and type I error for single-locus and region-based QTL mappings**. Power and type I error for three region-based QTL mappings: (A) combined analysis; (B) rare-variant-only analysis; and (C) common-SNP-only analysis. In each panel, the results of the single-locus QTL mapping are also provided for comparison. Red bars indicate that a QTL is identified by both single-locus and region-based QTL mappings. Blue hatched bars indicate that a QTL is identified only by the single-locus but not the region-based QTL mapping. Green hatched bars indicate that a QTL is identified only by the region-based but not the single-locus QTL mapping.

Our analyses were performed with knowledge of the underlying simulation model. Power and type I error for the single-locus and the three region-based QTL mappings for identification of the nine Q1-associated genes are shown in Figure 1. In general, for both single-locus and region-based QTL mappings, type I error was controlled under the prespecified nominal significance level of 0.05 for the nine Q1-associated genes. Power to detect the nine Q1-associated genes varied by the method of QTL mapping and the genes studied, reflecting the discrepancy in the effect sizes and the frequencies of rare variants and common SNPs within each of the nine Q1-associated genes in the simulation data set. *KDR* and *FLT1*, which were the top two genes in terms of total effect size, were successfully identified by all methods. *ELAVL4*, *ARNT*, and *HIF1A* were identified primarily by the analyses that used common SNP information. In contrast, *VEGFC* and *VEGFA* were identified primarily by the analyses that used rare variant information; *VEGFC* did not contain any common SNPs. The power to identify *FLT4* came primarily from the combined information provided by both common SNPs and rare variants. *HIF3A* was the most difficult gene to identify.

In a comparison of region-based and single-locus QTL mappings, the region-based QTL mappings often had greater power than the single-locus QTL mappings. The only counterexamples were the following: The single-locus analysis was more powerful than the combined analysis in identifying *ARNT* (a difference in power of 9%) and *HIF3A* (a difference in power of 8.2%) (Figure 1A); and the single-locus analysis was more powerful than the rare-variant-only analysis in identifying *FLT1* (a difference in power of 5%) (Figure 1B). These results can be explained by the fact that *ARNT* and *FLT1* contain several markers that have highly significant *p*-values. In a comparison of the three region-based analyses, the combined analysis generally performed better than the other two region-based analyses (Figure 1A). The rare-variant-only analysis showed very low power in detecting *ELAVL4* (2%), *ARNT* (2.3%), *HIF1A* (9.3%), and *HIF3A* (5.4%) (Figure 1B), and the common-SNP-only analysis showed very low power in detecting *VEGFC* (0%) and *VEGFA* (4.6%); *VEGFC* did not contain any common SNPs (Figure 1C). The integration of the results from the single-locus QTL mapping and the combined analysis results from the region-based QTL mapping provided competitive power and reasonable type I error compared with the results from the other QTL mappings. Similar results were also found when we conducted QTL mappings without a covariate adjustment or with an adjustment for age or smoking status alone (data not shown).

Pathway-based QTL mappings were evaluated by examining the power related to the detection of an association between Q1 and the VEGF pathway and by examining the type I error related to the detection of an association between Q4 and the VEGF pathway. We conducted the combined, rare-variant-only, and common-SNP-only analyses. The results showed that the power for the three analyses was close to 100% and that the type I error for the combined, rare-variant-only, and common-SNP-only analyses was 6.1%, 8.2%, and 5.8%, respectively. Pathways other than the VEGF pathway were not analyzed because we did not know whether or not they were associated with Q1.

We applied region-based QTL mappings to analyze all 24,487 common SNPs and rare variants within 3,205 genes for the first replication of 200 simulations. The analysis was finished within half an hour using a personal computer with an Intel E8400 CPU and two DDR2-800 2GB RAMs. Among the nine Q1-associated genes, the single-locus QTL mapping identified four Q1-associated genes, namely, *KDR*, *VEGFC*, *FLT1*, and *HIF1A*. The combined region-based analysis also identified *KDR*, *VEGFC*, *FLT1*, and *HIF1A* as well as two Q1-associated genes, *ELAVL4* and *VEGFA*.

## Discussion and conclusions

Region-based QTL mapping using a truncation product *p*-value method provides an alternative to single-locus QTL mapping. In comparison with single-locus QTL mapping, region-based QTL mapping has several strengths [4-6], as discussed in the Background section. We recommend using region-based QTL mapping with *p*-value combination unless the study region contains markers with strong marginal effects. Single-locus mapping is powerful enough to identify a QTL with a strong marginal effect. Among the three proposed region-based analyses, a combined analysis that uses information from both common SNPs and rare variants provides greater power than a rare-variant-only analysis or a common-SNP-only analysis. In a combined analysis, an alternative to the combined test statistic used in this study is the minimum test statistic of the rare-variant-only and common-SNP-only TPPS. However, our simulation results show that the multiplication test statistic is more powerful than the minimum test statistic (data not shown).

We suggest an integrated analysis of the single-locus method and the combined region-based method for QTL mapping of deep DNA sequencing data; this strategy provides a powerful way to identify QTLs with a well-controlled type I error. In addition, this procedure can be extended to examine the biological pathways or biological processes involved in complex disease. Combined pathway-based QTL mapping with the TPPS provides high power in the detection of associations between the VEGF pathway and Q1 and reasonable type I error in the detection of associations between the VEGF pathway and Q4.

## Competing interests

The authors declare that they have no competing interests.

## Authors’ contributions

HCY conceived of the study, participated in its design and coordination, developed statistical methods, and prepared the manuscript. CWC developed analysis software and analyzed the data with HCY. All authors read and approved the final manuscript.
